# Supplementary material for: Lipoate-binding proteins and specific lipoate-protein ligases in microbial sulfur oxidation reveal an atpyical role for an old cofactor
Source: eLife. 2018 Jul 13;7:e37439. doi: 10.7554/eLife.37439 (PMC6067878; doi:10.7554/eLife.37439)
Supplement: Figure 2—source data 1. [file elife-37439-fig2-data1.docx]

**Figure 2-source data 1. Occurrence of *hdr-*like, *lbpA* and *lbpA* maturation genes in genome-sequenced prokaryotes** Typical hdr-like gene clusters in established chemo- and photolithoautotrophic sulfur oxidizers have an *hdrC1B1A-hyp-hdrC2B2* arrangement. Genes that are immediately linked are highlighted in yellow. radSAM1 radical SAM domain-containing protein 1, lplA, lipoate:protein ligase, GGred, FAD-NAD-binding protein with some similarity to geranylgeranyl reductases. Genes that appear to be located in the same operon are highlighted in yellow. Genes that are located close to each other but not in the same transcriptional unit are marked in a lighter yellow. RISC, reduced inorganic sulfur compounds.

| **Organism/group** | ***hdr*-like genes** | ***lbpA* genes** | | ***radSAM1-lplA-GGred-radSAM2*** | | | **2. lplA gene** | | | **Canonical pathway (*lipAB, lipL, lipM*)** | | **Comments/ references** |
| --- | --- | --- | --- | --- | --- | --- | --- | --- | --- | --- | --- | --- |
| **BACTERIA** |  |  | |  | | |  | | |  | |  |
| **PROTEOBACTERIA** |  |  | |  | | |  | | |  | |  |
| **α-PROTEOBACTERIA** |  |  | |  | | |  | | |  | |  |
| ***Rhodobacterales*** |  |  | |  | | |  | | |  | |  |
| ***Rhodobacteraceae*** |  |  | |  | | |  | | |  | |  |
| *Defluviimonas* *indica* DSM 24802^T^ | SAMN05444006_102161-166 | SAMN05444006_102168 | | SAMN05444006_102157-154 | | | no | | | SAMN05444006_10595, SAMN05444006_1105 | | RISC oxidation (*Jiang et al., 2014*) |
| *Rhodobacter aestuarii* JA296^T^ (JCM 144887^T^) | BW967_RS14395-420 | BW967_RS14430 | | BW967_RS14355-370 | | | no | | | SAMN05421580_101623, SAMN05421580_103182 | | RISC oxidation negative(*Venkata Ramana et al., 2009*), isolated from estuarine microbial mat |
| *Rhodobacter* *sp*. SW2 | RSW2DRAFT_RS16425-16450 | RSW2DRAFT_RS16455 | | RSW2DRAFT_RS09280, 290-300 | | | RSW2DRAFT_RS16410 | | | RSW2DRAFT_RS05200, RSW2DRAFT_RS07695 | | Oxidizes FeS to sulfate (*Ehrenreich and Widdel, 1994*) |
| ***Rhizobales*** |  |  | |  | | |  | | |  | |  |
| ***Phyllobacteriaceae*** |  |  | |  | | |  | | |  | |  |
| *Hoeflea* sp. BRH_c9 | VR78_06515-06490 | VR78_06480 | | no | | | VR78_06530 | | | VR78_02800, no *lipB* | | Sulfur compound oxidation not reported, isolated from deep subsurface clay rock formation (*Bagnoud et al., 2016*) |
| ***Hyphomicrobiaceae*** |  |  | |  | | |  | | |  | |  |
| *Hyphomicrobium denitrificans* ATCC 51888^T^ | Hden_0689-0694 | Hden_0696 | | Hden_0683-0686 | | | no | | | Hden_2670, Hden_2234 | | Oxidizes dimethyl sulfide to sulfate (*Koch and Dahl, 2018*) |
| *Hyphomicrobium sp.* GJ21 | HYPGJ_30416-410 | HYPGJ_30408 | | HYPGJ_30421-418 | | | no | | | HYPGJ_31698, HYPGJ_40109 | | Sulfur compound oxidation not reported, isolated from activated sludge wastewater treatment plant (*Bringel et al., 2017*) |
| ***Sphingomonadales*** |  |  | |  | | |  | | |  | |  |
| ***Erythrobacteraceae*** |  |  | |  | | |  | | |  | |  |
| Altererythrobacter epoxidivorans CGMCC 1.7731^T^ | AMC99_00780-775 | AMC99_00773 | | AMC99_00785, no *GGred*, AMC99_00784-783 | | | no | | | AMC99_01196, AMC99_02058 | | Sulfur compound oxidation not reported, isolated from marine cold-seep sediment (*Kwon et al., 2007; Li et al., 2016*) |
| **β-PROTEOBACTERIA** |  |  | |  | | |  | | |  | |  |
| ***Sulfuricellales*** |  |  | |  | | |  | | |  | |  |
| ***Sulfuricellaceae*** |  |  | |  | | |  | | |  | |  |
| *Sulfuricella denitrificans* skB26^T^ (DSM 22764^T^) | SCD_RS09320-305^††^ | SCD_RS03980 | | SCD_RS03985-4000 | | | no | | | SCD_RS00530, SCD_RS00525 | | RISC oxidation (*Watanabe et al., 2012*) |
| **γ-PROTEOBACTERIA** |  |  | |  | | |  | | |  | |  |
| ***Acidithiobacillales*** |  |  | |  | | |  | | |  | |  |
| ***Acidithiobacillaceae*** |  |  | |  | | |  | | |  | |  |
| *Acidithiobacillus caldus* SM-1 (CGMCC 1.7296) | Atc_2352-2347 | Atc_2344, Atc_2346 | | Atc_2343-2340 | | | Atc_2337 | | | Atc_2379, Atc_2378 | | RISC oxidation (*You et al., 2011a*) |
| *Acidithiobacillus ferrivorans* SS3 (DSM 17398) | Acife_2476-2471 | Acife_2468, Acife_2470 | | Acife_2464-2467 | | | Acife_2461 | | | Acife_2503, Aceife_2502 | | RISC oxidation (*Hallberg et al., 2010*) |
| *Acidithiobacillus ferrooxidans* ATCC 23270^T^ | AFE_2555-2550 | AFE_2547, AFE_2549 | | AFE2543-2546 | | | AFE_2540 | | | Afe_2570, Afe_2569 | | RISC oxidation (*Kelly and Wood, 2000*) |
| *Acidithiobacillus ferrooxidans* ATCC 53993 | Lferr_2185-2178 » | Lferr_2175, Lferr_2177 | | Lferr2171-2174 | | | Lferr_2168 | | | Lferr_2200, Lferr_2199 | | RISC oxidation (*Kelly and Wood, 2000*) |
| *Acidithiobacillus thiooxidans* ATCC 19377^T^ | ATHIO_RS0101720-0101745 | ATHIO_RS0101750, RS0101760 | | ATHIO_RS0101765-775 | | | no | | | ATHIO_RS0101590, ATHIO_RS0101595 | | RISC oxidation (*Kelly and Wood, 2000*) |
| ***Thermithiobacillaceae*** |  |  | |  | | |  | | |  | |  |
| *Thermithiobacillus tepidarius* DSM 3134^T^ | G579_RS0105310-0105285 | G579_RS0105270, RS0105280 | | G579_RS0105265-5250 | | | G579_RS0105230 | | | G579_RS0102775 (*lipA*) G579_RS0109695 (*lipA*) G579_RS15480 (*lipB*) | | RISC oxidation (*Kelly and Wood, 2000*) |
| ***Chromatiales*** |  |  | |  | | |  | | |  | |  |
| ***Ectothiorhodospiraceae*** |  |  | |  | | |  | | |  | |  |
| *Ectothiorhodospira* sp. PHS-1 | ECTPHS_RS10575 -10600 | ECTPHS_RS10605, RS10615 | | ECTPHS_RS10620-10635 | | | no | | | ECTPHS_RS09075, ECTPHS_RS02465 | | RISC oxidation (*Kulp et al., 2008; Zargar et al., 2012*) |
| *Ectothiorhodospira marina* DSM 241^T^ | SAMN05444515_102233-2238 | SAMN05444515_102239, 102241 | | SAMN05444515_102242-102245 | | | SAMN05444515_10991 (weak) | | | SAMN05444515_1188, SAMN05444515_11261 | | RISC oxidation (*Imhoff, 2005*) |
| *Ectothiorhodospira haloalkaliphila* ATCC 51935^T^ | ECTHA_RS0104485-0104460 | ECTHA_RS0104445, RS0104455 | | ECTHA_RS0104440-0104425 | | | no | | | ECTHA_RS0115590, ECTHA_RS0109110 | | RISC oxidation (*Imhoff, 2005*) |
| *Ectothiorhodospira mobilis* DSM 4180 | SAMN05421721_10344-10349 | SAMN05421721_10350, 10352 | | SAMN05421721_10353-10356 | | | SAMN05421721_11133 (weak) | | | SAMN05421721_11419, SAMN05421721_12217 | | RISC oxidation (*Imhoff, 2005*) |
| *Ectothiorhodosinus mongolicus* M9^T^ (DSM 15479^T^) | B0B04_RS03070-03045 | B0B04_RS03040, RS03030 | | no | | | no | | | B0B04_RS04725, B0B04_RS07340 | | RISC oxidation (*Gorlenko et al., 2004*) |
| *Halorhodospira halochloris* str. A (DSM 1059^T^) | M911_11240-11215 | M911_11200, M911_11210 | | M911_11195-11180 | | | no | | | M911_01920, M911_03385 | | Sulfide oxidation (*Imhoff, 2005*) |
| *Acidihalobacter prosperus* V6 (DSM 14174) | BJI67_RS04060-85 | BJI67_RS04090, BJI67_RS04100 | | BJI67_RS04105-04120 | | | BJI67_RS04910 | | | BJI67_RS04410, BJI67_RS04405 | | RISC oxidation (*Khaleque et al., 2017*) |
| *Thioalkalivibrio nitratireducens* DSM 14787^T^ | TVNIR_3249-3244 | TVNIR_3241, TVNIR_3243 | | no | | | TVNIR_3231 (not the same operon) | | | TVNIR_2196, TVNIR_2197 | | RISC oxidation (*Sorokin et al., 2003*) |
| *Thioalkalivibrio* sp. K90mix | TK90_0632-0637 | TK90_0638, TK90_0640 | | TK90_0641-0644 | | | TK90_0648 | | | TK90_1966, TK90_1967 | | RISC oxidation (*Muyzer et al., 2011a*) |
| *Thioalkalivibrio sulfidophilus* HL-EbGr7 | Tgr7_2216-2211 | Tgr7_2208, Tgr7_2210 | | Tgr7_2207-2204 | | | Tgr7_2168 | | | Tgr7_2696, Tgr7_2704 | | RISC oxidation (*Muyzer et al., 2011a*) |
| *Thioalkalivibrio sp.* ALJ3 | C935_RS0110745-720 | C935_RS0110705, R0110715 | | C935_RS0110700-0110685 | | | no | | | C935_RS0107845, C935_RS0107850 | | RISC oxidation (*Muyzer et al., 2011b*) |
| *Thioalkalivibrio sp.* AKL11 | D574_RS0112030-005 | D574_RS0111990, 0112000 | | D574_RS0111985-0111970 | | | D574_RS0111950 | | | D574_RS0111650, D574_RS0111645 | | RISC oxidation (*Muyzer et al., 2011b*) |
| *Thioalkalivibrio sp.* ALMg13-2 | F618_RS0106210-185 | F618_RS0106170, 0106180 | | F618_RS0106165-0106150 | | | F618_RS0112570 | | | F618_RS0103420, F618_RS0103425 | | RISC oxidation (*Muyzer et al., 2011b*) |
| *Thiorhodospira sibirica* ATCC 700588^T^ | ThisiDRAFT_1539-34 | ThisiDRAFT_1533, 2312 | | ThisiDRAFT_0082, 0513, 1283, 1859 | | | no | | | ThisiDRAFT_0545, ThisiDRAFT_2181 | | Sulfide oxidation (*Bryantseva et al., 1999; Imhoff, 2005*) |
| *Thioalkalivibrio versutus* D301 | TVD_10650-10625 | TVD_10610, TVD_10620 | | TVD_10605-10590 | | | TVD_10570 | | | TVD_11020, TVD_11025 | | RISC oxidation (*Imhoff, 2005; Mu et al., 2016*) |
| *Thiohalospira halophila* DSM 15071^T^ | SAMN05660831_02553-557 | SAMN05660831_02558, 02560 | | SAMN05660831_02561-02564 | | | SAMN05660831_02568 | | | SAMN05660831_01616, SAMN05660831_01951 | | RISC oxidation (*Sorokin et al., 2008b*) |
| *Thiohalorhabdus denitrificans* HL 19 (DSM 15699^T^) | BLP36_RS08655-630 | BLP36_RS08615, RS08625 | | BLP36_RS08610-8595 | | | BLP36_RS08575 | | | BLP36_RS10220, BLP36_RS10225 | | RISC oxidation (*Sorokin et al., 2008a*) |
| **ACTINOBACTERIA** |  |  | |  | | |  | | |  | |  |
| **ACTINOBACTERIA** |  |  | |  | | |  | | |  | |  |
| ***Pseudonocardiales*** |  |  | |  | | |  | | |  | |  |
| ***Pseudonocardiaceae*** |  |  | |  | | |  | | |  | |  |
| *Saccharomonospora marina* XMU15 (DSM 45390^T^) | SACMADRAFT_RS09370-385* | | SACMADRAFT_RS09400, RS09445, RS09450 | | SACMADRAFT_RS09405-09415 no *GGred* | | | SACMADRAFT_RS28470 | | SACMADRAFT_RS07040, SACMADRAFT_RS07065, no *lipM,* no *lipL* | | Sulfur compound oxidation not reported, isolated from ocean sediment (*Klenk et al., 2012; Liu et al., 2010*)  linkage of *hdrBCA* with genes for CCG domain proteins and *etfBA* and Lbp biosynthesis genes |
| ***Corynebacteriales*** |  |  | |  | | |  | | |  | |  |
| ***Gordoniaceae*** |  |  | |  | | |  | | |  | |  |
| *Gordonia rhizosphera* NBRC 16068^T^ | GORHZ_RS13510-13495* | GORHZ_RS13480, GORHZ_RS18175, 18180 | | GORHZ_RS13475, RS13470, no *GGred*, GORHZ_RS05265 | | | no | | | GORHZ_RS00170, GORHZ_RS00165, no *lipM*, no *lipL* | | Sulfur oxidation not reported, isolated from Mangrove rhizosphere (*Takeuchi and Hatano, 1998*), genes on different contigs |
| **ACIDIMICROBIA** |  |  | | | |  | | |  | |  | |
| ***Acidimicrobiales*** |  |  | |  | | |  | | |  | |  |
| ***Acidimicrobiaceae*** |  |  | |  | | |  | | |  | | Oxidation of FeS_2_, reduced sulfur compounds not oxidized (*Johnson et al., 2009*) |
| *Ferrithrix thermotolerans* DSM 19514^T^ | SAMN02745225_01749-01751* | SAMN02745225_01759, 01758 | | no | | | no | | | SAMN02745225_01216, (*lipAB* fusion), no *lipM*, no *lipL* | | Linkage of *hdrBCA* with genes for CCG domain proteins and *etfBA* |
| *Acidimicrobium ferrooxidans* DSM 10331^T^ | Afer_0964-67* | Afer_0973, Afer_0974, Afer_1055 | | Afer_1054-1052, no *GGred*, | | | no | | | Afer_0719 (*lipAB* fusion), no *lipM*, no *lipL* | | Oxidation of FeS_2_ (*Clark and Norris, 1996; Clum et al., 2009*), Linkage of *hdrBCA* with genes for CCG domain proteins and *etfBA* |
| **FIRMICUTES** |  |  | |  | | |  | | |  | |  |
| **CLOSTRIDIA** |  |  | |  | | |  | | |  | |  |
| ***Clostridiales*** |  |  | |  | | |  | | |  | |  |
| **Family XVII. *Incertae Sedis*** |  |  | |  | | |  | | |  | |  |
| *Sulfobacillus acidophilus* DSM 10332^T^ | Sulac_1384-87* | Sulac_1389, Sulac_1390, Sulac_2875 | | Sulac_2874 (*lplA*), 2876 (*lplA*), 2877 (*radSAM*), 2878 (*radSAM*), no *GGred* | | | Sulac_1391 | | | Sulac_3479, Sulac_3478, no *lipM*,, no *lipL* | | Oxidation of mineral sulfides, RISC oxidation (*Watling et al., 2008*), linkage of *hdrBCA* with genes for CCG domain proteins, *etfBA* and one *lplA*-like gene |
| *Sulfobacillus acidophilus* TPY | TPY_3531-28* | TPY_3526, TPY_3525, TPY_0765 | | TPY_0766 (*lplA*), 0764 (*lplA*), 0763 (*radSAM*), 0762 (*radSAM*), no *GGred* | | | TPY_3524 | | | TPY_3785, TPY_3784, no *lipM*, *no lipL* | | Oxidation of mineral sulfides, RISC oxidation (*Guo et al., 2016*), linkage of *hdrBCA* with genes for CCG domain proteins, *etfBA* and one *lplA*-like gene |
| *Sulfobacillus thermosulfidooxidans* DX | BFX05_RS00130-145* | BFX05_RS00170, RS00175, BFX05_RS14350 | | BFX05_RS14345 (*lplA*), RS14355 (*lplA*) BFX05_RS14360 (*radSAM*), BFX05_RS14365 (*radSAM*) | | | BFX05_RS00180 | | | BFX05_RS03940, BFX05_RS03935, no *lipM*  no *lipL* | | Oxidation of mineral sulfides, RISC oxidation (*Zhang et al., 2017*), linkage of *hdrBCA* with genes for CCG domain proteins, *etfBA* and one *lplA*-like gene |
| **BACILLI** |  |  | |  | | |  | | |  | |  |
| ***Bacilliales*** |  |  | |  | | |  | | |  | |  |
| ***Alicyclobacillaceae*** |  |  | |  | | |  | | |  | |  |
| *Kyrpidia tusciae* DSM 2912^T^ | Btus_2498-95* | Btus_2489, Btus_2490, Btus_2481 | | Btus_2482 (*lplA*), Btus_2480 (*lplA*), Btus_2479 (*radSAM*), Btus_2478 *(radSAM*) | | | Btus _2488 (ligase) | | | Btus_1312, no *lipB* Btus_1766 (*lipM* ), no *lipL* | | Sulfur oxidation not reported, isolated from solfatara (*Bonjour and Aragno, 1984; Klenk et al., 2011*), Two clusters of the Sulfobacilli are linked |
| **CHLOROBI** |  |  | |  | | |  | | |  | |  |
| **CHLOROBIA** |  |  | |  | | |  | | |  | |  |
| ***Chlorobiales*** |  |  | |  | | |  | | |  | |  |
| ***Chlorobiaceae*** |  |  | |  | | |  | | |  | |  |
| *Chloroherpeton thalassium* ATCC 35110^T^ | Ctha_0138-0136 # | Ctha_0140, Ctha_0129 | | Ctha_0128 (*lplA*), Ctha 127 (*radSAM*), Ctha_0126 (*radSAM)*, Ctha_125 (*lplA*), Ctha_124 (*radSAM*), no *GGred* | | | no | | | Ctha_2201, Ctha_0430 | | Oxidation of sulfide (*Bonjour and Aragno, 1984*), possible operon is broken by Ctha_0135, linkage of *hdrBCA* with genes for CCG domain proteins and *etfBA* |
| **CHLOROFLEXI** |  |  | |  | | |  | | |  | |  |
| **THERMOMICROBIA** |  |  | |  | | |  | | |  | |  |
| ***Thermomicrobiales*** |  |  | |  | | |  | | |  | |  |
| ***Thermomicrobiaceae*** |  |  | |  | | |  | | |  | |  |
| *Thermomicrobium roseum* DSM 5159^T^ | trd_0155-52* | trd_0144, trd_143, trd_0157 | | no | | | Trd_0142 (*lplA*) | | | trd_0168, trd_0170 | | Sulfur oxidation not reported, isolated from Yellowstone hot spring (*Wu et al., 2009*), linkage of *hdrBCA* with genes for CCG domain proteins, *etfBA* and one *lplA*-like gene |
| **AQUIFICAE** |  |  | |  | | |  | | |  | |  |
| **AQUIFICAE** |  |  | |  | | |  | | |  | |  |
| ***Aquificales*** |  |  | |  | | |  | | |  | |  |
| ***Aquificaceae*** |  |  | |  | | |  | | |  | |  |
| *Aquifex aeolicus* VF5 | aq_391-400 | aq_402 | | aq_403, aq_1638, aq_121, aq_1958 | | | no | | | aq_1355, no *lipB* | | RISC oxidation (*Gupta and Lali, 2013*) |
| *Hydrogenivirga* sp. 128-5-R1-1 | HG1285_RS14290-14265 | HG1285_RS14255 | | HG1285_RS14250, HG1285_06515, 15109, 16345 | | | no | | | HG1285_06045, HG1285_04243 (both *lipA*), no *lipB* | | RISC oxidation (*Gupta and Lali, 2013*) |
| *Hydrogenobacter thermophilus* TK-6^T^ (DSM 6534^T^) | HTH_1882-HTH_1878 | HTH_1874, HTH_1877 | | HTH_1873, HTH_1603, 1747 HTH_1871 | | | no | | | HTH_0701, HTH_0579 (both *lipA*), no *lipB* | | RISC oxidation (*Gupta and Lali, 2013*) |
| *Hydrogenobaculum* sp. HO | HydHO_1077-1072 | HydHO_1068, HydHO_1071 | | HydHO_1066, HydHO_0926, HydHO_0802, HydHO_0584 | | | no | | | no *lipA*, no *lipB* | | RISC oxidation (*Gupta and Lali, 2013; Romano et al., 2013*) |
| *Hydrogenobaculum* sp. Y04AAS1 | HY04AAS1_RS05470-05445 | HY04AAS1_RS05425, HY04AAS1_RS05440 | | HY04AAS1_RS05415, RS04710, RS04065, RS02975 | | | no | | | no *lipA*, no *lipB* | | RISC oxidation (*Gupta and Lali, 2013; Romano et al., 2013*) |
| *Thermocrinis albus* DSM 14484^T^ | THAL_RS06855-06830 | THAL_RS06810, THAL_RS06825 | | THAL_RS06805, RS01130, RS04395, RS01575 | | | no | | | THAL_RS05455, no *lipB* | | RISC oxidation (*Gupta and Lali, 2013*) |
| *Thermocrinis jamiesonii* GBS^T^ (DSM 27162^T^) | K217_RS0105820-0105845 | K217_RS0105850, RS0105865 | | K217_RS0105870, RS0101120 RS0100395, RS0104270 | | | no | | | K217_RS0107180, no *lipB* | | RISC oxidation (*Ganji et al., 2016; Gupta and Lali, 2013*) |
| *Thermocrinis minervae* DSM 19557^T^ | SAMN05444391_ 1193-1188 | SAMN05444391_1184, SAMN05444391_1187 | | SAMN05444391_1183, _1443, _1281, _0147 | | | no | | | SAMN05444391_1343, SAMN05444391_0326 (both *lipA*), no *lipB* | | RISC oxidation (*Caldwell et al., 2010; Gupta and Lali, 2013*) |
| **ARCHAEA** |  |  | |  | | |  | | |  | |  |
| **CRENARCHAEOTA** |  |  | |  | | |  | | |  | |  |
| ***Sulfolobales*** |  |  | |  | | |  | | |  | |  |
| ***Sulfolobaceae*** |  |  | |  | | |  | | |  | |  |
| *Acidianus hospitalis* W1 | Ahos_1696-1691 | Ahos_1686, Ahos_1684 | | Ahos_1681, Ahos_1682, no *GGred,* Ahos_1680 | | | Ahos_1685 | | | no *lipA*, no *lipB*,  archaeal LplA-LplB system:  Ahos_0168, Ahos_0169 | | Oxidation of sulfur (*You et al., 2011b*), *lbp* gene(s) in vicinity but not in same operon as *hdr* genes and biosynthetic genes |
| *Metallosphaera cuprina* Ar-4^T^ (JCM 15769^T^) | Mcup_0684-0689 | Mcup_0662 | | Mcup_0672, Mcup_0671, no *GGred,* Mcup_0729 | | | Mcup_0767 | | | no *lipA,* no *lipB,* archaeal LplA-LplB system: no, Mcup_0731 lipoate:protein ligase, Mcup_0730 radSAM, Mcup_0729 radSAM, | | Oxidation of tetrathionate, FeS and FeS_2_ (*Liu et al., 2011*) *lbp* gene(s) in vicinity but not in same operon as *hdr* genes and biosynthetic genes |
| *Metallosphaera sedula* DSM 5348^T^ | Msed_1547-1542 | Msed_1570 | | Msed_1559, Msed_1560, no *GGred,* Msed_1558 | | | Msed_1480 | | | no *lipA,* no *lipB,* archaeal LplA-LplB system: no Msed_1506 lipoate:protein ligase, Msed_1507 radSAM, Msed_1508 radSAM | | Oxidation of sulfur and metal sulfides (*Auernik et al., 2008*) *lbp* gene(s) in vicinity but not in same operon as *hdr* genes and biosynthetic genes |
| *Sulfolobus acidocaldarius* DSM 639^T^ | Saci_0334/0329-0325 | Saci_0349, Saci_0350 | | Saci_0344, Saci_0345, no *GGred,* Saci_0343 | | | Saci_0351 | | | Saci_0309 (lipoyl synthase) no *lipB*  Archaeal LplA-LplB system: Saci_0307, Saci_0310, no *lplB* | | Sulfur oxidation of lab strain unclea (*Brock et al., 1972; Chen et al., 2005; Zillig et al., 1994*) *lbp* gene(s) in vicinity but not in same operon as *hdr* genes and biosynthetic genes |
| *Sulfolobus “islandicus”* M.14.25 | M1425_1101-1096 | M1425_1122 | | M1425_1113, M1425_1114, no *GGred,* M1425_1112 | | | M1425_1159 | | | M1425_2212, no *lipB* Archaeal LplA-LplB system: M1425_2214, no *lplB*, archaeal LipA: M1425_2212, M1425_2211, M1425_0822. | | Sulfur oxidation of lab strain unclear (*Reno et al., 2009; Zillig et al., 1994*) *lbp* gene(s) in vicinity but not in same operon as *hdr* genes and biosynthetic genes |
| *Sulfolobus solfataricus* P2 (DSM 1617) | SSO1127-1135 | SSO1105 | | SSO1114, SSO1112, no *GGred,* SSO1115 | | | SSO1060 | | | SSO3158, no *lipB,* Archaeal LplA-LplB system: SSO3157, no *lplB,* archaeal *lipA* SSO3158  SSO3157 (*lplA-1*), SSO3158 (LipA), SSO3159 (*lplA-2*) | | Sulfur oxidation of lab strain unclear (*Zillig et al., 1994; Zillig et al., 1980*) *lbp* gene(s) in vicinity but not in same operon as *hdr* genes and biosynthetic genes |
| *Sulfolobus tokodaii* str. 7 (DSM 16993^T^) | STK_18750-18700 | STK_18940, STK_18950 | | STK_18870, STK_18880, no *GGred,* STK_18860 | | | STK_18960 | | | No lipoate:protein ligases or lipoyl synthases | | Slow oxidation of sulfur (*Suzuki et al., 2002*), *lbp* gene(s) in vicinity but not in same operon as *hdr* genes and biosynthetic genes |

^a^Genomes were analysed by BLAST searches using the resources provided by Integrated Microbial Genomes (DOE Joint genomes Institute, <http://img.jgi.doe.gov>) and GenBank (<http://www.ncbi.nml.nih.gov>. Baits: Hdr-like proteins from *Acidithiobacillus caldus* SM1: HdrC1, Atc_2352; HdrB1, Atc_2351; HdrA, Atc_2350; Hyp, Atc_2349;, HdrC2, Atc_2348; HdrB2, Atc_2347; lipoate-binding proteins and their biosynthesis from *A. caldus*, LbpA1, Atc_2346; LbpA2, Atc_2344; radical SAM protein 1, Atc_2343; single domain LplA (Atc_2342), geranylgeranyl reductase like FAD-NAD-binding protein, Atc_2341; radical SAM protein 2, Atc_2340; second LplA-like protein, Atc_2337; LipA (AAA66345) and LipB (AAA66342) from *E. coli* str. K-12 substr. W3110. Actinobacteria and Firmicutes were also searched for LipM (BSU24530) and LipL (BSU37640) from *Bacillus subtilis* subsp. s*ubtilis* str. 168. Archaea were checked for occurrence of archaeal LplAB from *Thermoplasma acidophilum* (*Christensen and Cronan, 2009*).

**References**

Auernik K, Maezato Y, Blum PH, Kelly RM. 2008. The genome sequence of the metal-mobilizing, extremely thermoacidophilic archaeon *Metallosphaera sedula* provides insights into bioleaching-associated metabolism. *Applied and Environmental Microbiology* **74**:682-692. doi: 10.1128/Aem.02019-07, PMID: 18083856

Bagnoud A, Chourey K, Hettich RL, de Bruijn I, Andersson AF, Leupin OX, Schwyn B, Bernier-Latmani R. 2016. Reconstructing a hydrogen-driven microbial metabolic network in Opalinus Clay rock. *Nature Communications* **7**:12770. doi: 10.1038/ncomms12770, PMCID: PMC5067608

Bonjour F, Aragno M. 1984. *Bacillus tusciae*, a new species of thermoacidophilic, facultatively chemolithoautotrophic, hydrogen oxidizing sporeformer from a geothermal area. *Archives of Microbiology* **139**:397-401. doi: 10.1007/Bf00408386

Bringel F, Postema CP, Mangenot S, Bibi-Triki S, Chaignaud P, Farhan Ul Haque M, Gruffaz C, Hermon L, Louhichi Y, Maucourt B, Muller EEL, Nadalig T, Lajus A, Rouy Z, Medigue C, Barbe V, Janssen DB, Vuilleumier S. 2017. Genome sequence of the dichloromethane-degrading bacterium *Hyphomicrobium* sp. strain GJ21. *Genome Announcements* **5**:e00622-17. doi: 10.1128/genomeA.00622-17, PMCID: PMC5532824

Brock TD, Brock KM, Belly RT, Weiss RL. 1972. *Sulfolobus*: a new genus of sulfur-oxidizing bacteria living at low pH and high temperatures. *Archiv für Mikrobiologie* **84**:54-68. PMID: 4559703

Bryantseva IA, Gorlenko VM, Kompantseva EI, Imhoff JF, Sling J, Mityushina L. 1999. *Thiorhodospira sibirica* gen. nov., sp. nov., a new alkaliphilic purple sulfur bacterium from a Siberian soda lake. *International Journal of Systematic Bacteriology* **49**:697-703. doi: 10.1099/00207713-49-2-697, PMID: 10319493

Caldwell SL, Liu Y, Ferrera I, Beveridge T, Reysenbach AL. 2010. *Thermocrinis minervae* sp. nov., a hydrogen- and sulfur-oxidizing, thermophilic member of the Aquificales from a Costa Rican terrestrial hot spring. *International Journal of Systematic and Evolutionary Microbiology* **60**:338-43. doi: 10.1099/ijs.0.010496-0, PMID: 19651724

Chen L, Brugger K, Skovgaard M, Redder P, She Q, Torarinsson E, Greve B, Awayez M, Zibat A, Klenk HP, Garrett RA. 2005. The genome of *Sulfolobus acidocaldarius*, a model organism of the Crenarchaeota. *Journal of Bacteriology* **187**:4992-9. doi: 10.1128/JB.187.14.4992-4999.2005, PMCID: PMC1169522

Christensen QH, Cronan JE. 2009. The *Thermoplasma acidophilum* LplA-LplB complex defines a new class of bipartite lipoate-protein ligases. *Journal of Biological Chemistry* **284**:21317-21326. doi: 10.1074/jbc.M109.015016, PMCID: PMC2755856

Clark DA, Norris PR. 1996. *Acidimicrobium ferrooxidans* gen. nov., sp. nov : mixed-culture ferrous iron oxidation with *Sulfobacillus* species. *Microbiology* **142**:785-790. doi: 10.1099/00221287-142-4-785,

Clum A, Nolan M, Lang E, Glavina Del Rio T, Tice H, Copeland A, Cheng JF, Lucas S, Chen F, Bruce D, Goodwin L, Pitluck S, Ivanova N, Mavrommatis K, Mikhailova N, Pati A, Chen A, Palaniappan K, Goker M, Spring S et al. 2009. Complete genome sequence of *Acidimicrobium ferrooxidans* type strain (ICP). *Standards in Genomic Sciences* **1**:38-45. doi: 10.4056/sigs.1463, PMCID: PMC3035218

Ehrenreich A, Widdel F. 1994. Anaerobic oxidation of ferrous iron by purple bacteria, a new type of phototrophic metabolism. *Applied and Environmental Microbiology* **60**:4517-4526. PMCID: PMC202013

Ganji R, Murugapiran SK, Ong JC, Manoharan N, Huntemann M, Clum A, Pillay M, Palaniappan K, Varghese N, Mikhailova N, Stamatis D, Reddy TB, Ngan CY, Daum C, Duffy K, Shapiro N, Markowitz V, Ivanova N, Kyrpides N, Woyke T et al. 2016. High-quality draft genome sequence of *Thermocrinis jamiesonii* GBS1^T^ isolated from Great Boiling Spring, Nevada. *Genome Announcements* **4**:e01112-16. doi: 10.1128/genomeA.01112-16, PMCID: PMC5073254

Gorlenko VM, Bryantseva IA, Panteleeva EE, Tourova TP, Kolganova TV, Makhneva ZK, Moskalenko AA. 2004. *Ectothiorhodosinus mongolicum* gen. nov., sp. nov., a new purple bacterium from a soda lake in Mongolia. *Microbiology* **73**:66-73. PMID: 15074045

Guo W, Zhang H, Zhou W, Wang Y, Zhou H, Chen X. 2016. Sulfur metabolism pathways in *Sulfobacillus acidophilus* TPY, a gram-positive moderate thermoacidophile from a hydrothermal vent. *Frontiers in Microbiology* **7**:1861. doi: 10.3389/fmicb.2016.01861, PMCID: PMC5114278

Gupta RS, Lali R. 2013. Molecular signatures for the phylum Aquificae and its different clades: proposal for division of the phylum Aquificae into the emended order *Aquificales*, containing the families *Aquificaceae* and *Hydrogenothermaceae*, and a new order *Desulfurobacteriales* ord. nov., containing the family *Desulfurobacteriaceae*. *Antonie van Leeuwenhoek* **104**:349-68. doi: 10.1007/s10482-013-9957-6, PMID: 23812969

Hallberg KB, González-Toril E, Johnson DB. 2010. *Acidithiobacillus ferrivorans*, sp. nov.; facultatively anaerobic, psychrotolerant iron-, and sulfur-oxidizing acidophiles isolated from metal mine-impacted environments. *Extremophiles* **14**:9-19. doi: 10.1007/s00792-009-0282-y, PMID: 19787416

Imhoff JF (2005) Family II. Ectothiorhodospiraceae Imhoff 1984b, 339 VP In: Brenner DJ, Krieg NR, Staley JT, Garrity GM (editors). *Bergey's manual of systematic bacteriology.* New York: Springer, pp41-57.

Jiang L, Long M, Shao Z. 2014. Draft genome sequence of *Defluviimonas indica* strain 20V17^T^, Isolated from a deep-sea hydrothermal vent environment in the Southwest Indian Ocean. *Genome Announcements* **2**:e00479-14. doi: 10.1128/genomeA.00479-14, PMCID: PMC4047444

Johnson DB, Bacelar-Nicolau P, Okibe N, Thomas A, Hallberg KB. 2009. *Ferrimicrobium acidiphilum* gen. nov., sp. nov. and *Ferrithrix thermotolerans* gen. nov., sp. nov.: heterotrophic, iron-oxidizing, extremely acidophilic actinobacteria. *International Journal of Systematic and Evolutionary Microbiology* **59**:1082-9. doi: 10.1099/ijs.0.65409-0, PMID: 19406797

Kelly DP, Wood AP. 2000. Reclassification of some species of *Thiobacillus* to the newly designated genera *Acidithiobacillus* gen. nov., *Halothiobacillus* gen. nov. and *Thermithiobacillus* gen. nov. *International Journal of Systematic and Evolutionary Microbiology* **50**:511-516. doi: 10.1099/00207713-50-2-511, PMID: 10758854

Khaleque HN, Ramsay JP, Murphy RJ, Kaksonen AH, Boxall NJ, Watkin EL. 2017. Draft genome sequence of the acidophilic, halotolerant, and iron/sulfur-oxidizing *Acidihalobacter prosperus* DSM 14174 (Strain V6). *Genome Announcements* **5**:e01469-16. doi: 10.1128/genomeA.01469-16, PMCID: PMC5255921

Klenk HP, Lapidus A, Chertkov O, Copeland A, Del Rio TG, Nolan M, Lucas S, Chen F, Tice H, Cheng JF, Han C, Bruce D, Goodwin L, Pitluck S, Pati A, Ivanova N, Mavromatis K, Daum C, Chen A, Palaniappan K et al. 2011. Complete genome sequence of the thermophilic, hydrogen-oxidizing *Bacillus tusciae* type strain (T2) and reclassification in the new genus, *Kyrpidia* gen. nov. as *Kyrpidia tusciae* comb. nov. and emendation of the family Alicyclobacillaceae da Costa and Rainey, 2010. *Standards in Genomic Sciences* **5**:121-34. doi: 10.4056/sigs.2144922, PMCID: PMC3236038

Klenk HP, Lu M, Lucas S, Lapidus A, Copeland A, Pitluck S, Goodwin LA, Han C, Tapia R, Brambilla EM, Potter G, Land M, Ivanova N, Rohde M, Goker M, Detter JC, Li WJ, Kyrpides NC, Woyke T. 2012. Genome sequence of the ocean sediment bacterium *Saccharomonospora marina* type strain (XMU15^T^). *Standards in Genomic Sciences* **6**:265-75. doi: 10.4056/sigs.2655905, PMCID: PMC3387791

Koch T, Dahl C. 2018. A novel bacterial sulfur oxidation pathway provides a new link between the cycles of organic and inorganic sulfur compounds. *ISME Journal* **submitted**doi:

Kulp TR, Hoeft SE, Asao M, Madigan MT, Hollibaugh JT, Fisher JC, Stolz JF, Culbertson CW, Miller LG, Oremland RS. 2008. Arsenic(III) fuels anoxygenic photosynthesis in hot spring biofilms from Mono Lake, California. *Science* **321**:967-970. doi: 10.1126/science.1160799, PMID: 18703741

Kwon KK, Woo JH, Yang SH, Kang JH, Kang SG, Kim SJ, Sato T, Kato C. 2007. *Altererythrobacter epoxidivorans* gen. nov., sp. nov., an epoxide hydrolase-active, mesophilic marine bacterium isolated from cold-seep sediment, and reclassification of *Erythrobacter luteolus* Yoon et al. 2005 as *Altererythrobacter luteolus* comb. nov. *International Journal of Systematic and Evolutionary Microbiology* **57**:2207-11. doi: 10.1099/ijs.0.64863-0, PMID: 17911284

Li ZY, Wu YH, Huo YY, Cheng H, Wang CS, Xu XW. 2016. Complete genome sequence of a benzo[a]pyrene-degrading bacterium *Altererythrobacter epoxidivorans* CGMCC 1.7731^T^. *Marine Genomics* **25**:39-41. doi: 10.1016/j.margen.2015.11.009

Liu LJ, You XY, Zheng H, Wang S, Jiang CY, Liu SJ. 2011. Complete genome sequence of *Metallosphaera cuprina*, a metal sulfide-oxidizing archaeon from a hot spring. *Journal of Bacteriology* **193**:3387-3388. doi: 10.1128/JB.05038-11, PMCID: PMC3133273

Liu Z, Li Y, Zheng LQ, Huang YJ, Li WJ. 2010. *Saccharomonospora marina* sp. nov., isolated from an ocean sediment of the East China Sea. *International Journal of Systematic and Evolutionary Microbiology* **60**:1854-7. doi: 10.1099/ijs.0.017038-0, PMID: 19767358

Mu T, Zhou J, Yang M, Xing J. 2016. Complete genome sequence of *Thioalkalivibrio versutus* D301 isolated from Soda Lake in northern China, a typical strain with great ability to oxidize sulfide. *Journal of Biotechnology* **227**:21-22. doi: 10.1016/j.jbiotec.2016.04.019, PMID: 27080450

Muyzer G, Sorokin DY, Mavromatis K, Lapidus A, Clum A, Ivanova N, Pati A, D'Haeseleer P, Woyke T, Kyrpides NC. 2011a. Complete genome sequence of "*Thioalkalivibrio sulfidophilus*" HL-EbGr7. *Standards in Genomic Sciences* **4**:23-35. doi: 10.4056/sigs.1483693, PMID: 21475584

Muyzer G, Sorokin DY, Mavromatis K, Lapidus A, Foster B, Sun H, Ivanova N, Pati A, D'Haeseleer P, Woyke T, Kyrpides NC. 2011b. Complete genome sequence of *Thioalkalivibrio* sp. K90mix. *Standards in Genomic Sciences* **5**:doi:10.4056/sigs.2315092. doi: 10.4056/sigs.2315092, PMCID: PMC3368412

Reno ML, Held NL, Fields CJ, Burke PV, Whitaker RJ. 2009. Biogeography of the *Sulfolobus islandicus* pan-genome. *Proceedings of the National Academy of Sciences of the United States of America* **106**:8605-10. doi: 10.1073/pnas.0808945106, PMCID: PMC2689034

Romano C, D'Imperio S, Woyke T, Mavromatis K, Lasken R, Shock EL, McDermott TR. 2013. Comparative genomic analysis of phylogenetically closely related *Hydrogenobaculum* sp. isolates from Yellowstone National Park. *Applied and Environmental Microbiology* **79**:2932-43. doi: 10.1128/AEM.03591-12, PMCID: PMC3623155

Sorokin DY, Tourova TP, Galinski EA, Muyzer G, Kuenen JG. 2008a. *Thiohalorhabdus denitrificans* gen. nov., sp. nov., an extremely halophilic, sulfur-oxidizing, deep-lineage gammaproteobacterium from hypersaline habitats. *International Journal of Systematic and Evolutionary Microbiology* **58**:2890-7. doi: 10.1099/ijs.0.2008/000166-0, PMID: 19060078

Sorokin DY, Tourova TP, Muyzer G, Kuenen GJ. 2008b. *Thiohalospira halophila* gen. nov., sp. nov. and *Thiohalospira alkaliphila* sp. nov., novel obligately chemolithoautotrophic, halophilic, sulfur-oxidizing gammaproteobacteria from hypersaline habitats. *International Journal of Systematic and Evolutionary Microbiology* **58**:1685-92. doi: 10.1099/ijs.0.65654-0, PMID: 18599717

Sorokin DY, Tourova TP, Sjollema KA, Kuenen JG. 2003. *Thialkalivibrio nitratireducens* sp. nov., a nitrate-reducing member of an autotrophic denitrifying consortium from a soda lake. *International Journal of Systematic and Evolutionary Microbiology* **53**:1779-1783. doi: 10.1099/ijs.0.02615-0, PMID: 14657104

Suzuki T, Iwasaki T, Uzawa T, Hara K, Nemoto N, Kon T, Ueki T, Yamagishi A, Oshima T. 2002. *Sulfolobus tokodaii* sp. nov. (f. *Sulfolobus* sp. strain 7), a new member of the genus *Sulfolobus* isolated from Beppu Hot Springs, Japan. *Extremophiles* **6**:39-44. PMID: 11878560

Takeuchi M, Hatano K. 1998. *Gordonia rhizosphera* sp. nov. isolated from the mangrove rhizosphere. *International Journal of Systematic and Evolutionary Microbiology* **48**:907-912. doi: 10.1099/00207713-48-3-907, PMID: 9734045

Venkata Ramana V, Anil Kumar P, Srinivas TN, Sasikala C, Ramana Ch V. 2009. *Rhodobacter aestuarii* sp. nov., a phototrophic alphaproteobacterium isolated from an estuarine environment. *International Journal of Systematic and Evolutionary Microbiology* **59**:1133-6. doi: 10.1099/ijs.0.004507-0, PMID: 19406806

Watanabe T, Kojima H, Fukui M. 2012. Draft genome sequence of psychotolerant sulfur-oxidizing bacterium, *Sulfuricella denitrificans* skB26, and proteomic insights into cold adaptations. *Applied and Environmental Microbiology* **78**:6545-6549. doi: 10.1128/AEM.01349-12, PMID: 22773644

Watling HR, Perrot FA, Shiers DW. 2008. Comparison of selected characteristics of *Sulfobacillus* species and review of their occurrence in acidic and bioleaching environments. *Hydrometallurgy* **93**:57-65. doi: 10.1016/j.hydromet.2008.03.001,

Wu D, Raymond J, Wu M, Chatterji S, Ren Q, Graham JE, Bryant DA, Robb F, Colman A, Tallon LJ, Badger JH, Madupu R, Ward NL, Eisen JA. 2009. Complete genome sequence of the aerobic CO-oxidizing thermophile *Thermomicrobium roseum*. *PLoS One* **4**:e4207. doi: 10.1371/journal.pone.0004207, PMCID: PMC2615216

You XY, Guo X, Zheng HJ, Zhang MJ, Liu LJ, Zhu YQ, Zhu B, Wang SY, Zhao GP, Poetsch A, Jiang CY, Liu SJ. 2011a. Unraveling the *Acidithiobacillus caldus* complete genome and its central metabolisms for carbon assimilation. *Journal of Genetics and Genomics* **38**:243-52. doi: 10.1016/j.jgg.2011.04.006, PMID: 21703548

You XY, Liu C, Wang SY, Jiang CY, Shah SA, Prangishvili D, She Q, Liu SJ, Garrett RA. 2011b. Genomic analysis of *Acidianus hospitalis* W1 a host for studying crenarchaeal virus and plasmid life cycles. *Extremophiles* **15**:487-97. doi: 10.1007/s00792-011-0379-y, PMCID: PMC3119797

Zargar K, Conrad A, Bernick DL, Lowe TM, Stolc V, Hoeft S, Oremland RS, Stolz J, Saltikov CW. 2012. ArxA, a new clade of arsenite oxidase within the DMSO reductase family of molybdenum oxidoreductases. *Environmental Microbiology* **14**:1635-45. doi: 10.1111/j.1462-2920.2012.02722.x, PMID: 22404962

Zhang X, Liu X, Liang Y, Guo X, Xiao Y, Ma L, Miao B, Liu H, Peng D, Huang W, Zhang Y, Yin H. 2017. Adaptive evolution of extreme acidophile *Sulfobacillus thermosulfidooxidans* potentially driven by horizontal gene transfer and gene loss. *Applied and Environmental Microbiology* **83**:e03098-16. doi: 10.1128/AEM.03098-16, PMCID: PMC5359484

Zillig W, Kletzin A, Schleper C, Holz I, Janekovic D, Hain J, Lanzendörfer M, Kristjansson JK. 1994. Screening for Sulfolobales, their plasmids and their viruses in Icelandic solfataras. *Systematic and Applied Microbiology* **16**:609-628.

Zillig W, Stetter KO, Wunder S, Schulz W, Priess H, Scholz I. 1980. The *Sulfolobus*-"*Caldariella*" group: taxonomy on the basis of the structure of DNA-dependent RNA polymerases. *Archives of Microbiology* **125**:259-269. doi: 10.1007/Bf00446886,
