# Supplementary material for: Lipoate-binding proteins and specific lipoate-protein ligases in microbial sulfur oxidation reveal an atpyical role for an old cofactor
Source: eLife. 2018 Jul 13;7:e37439. doi: 10.7554/eLife.37439 (PMC6067878; doi:10.7554/eLife.37439)
Supplement: Figure 3—source data 1. [file elife-37439-fig3-data1.docx]

**Figure 3-source data 1.** List of source organisms, locus tags and accessions for the LbpA and GcvH proteins considered in phylogenetic trees shown in Figure 3 and Figure 3-figure supplement 1.

| ***Organism*** | ***locus tag*** | ***accession*** |
| --- | --- | --- |
| *Acidianus hospitalis* W1 | Ahos_1686 | AEE94563 |
| *Acidihalobacter prosperus* V6 (DSM 14174) | BJI67_RS04090 | WP_070071950 |
| *Acidihalobacter prosperus* V6 (DSM 14174) | BJI67_RS04100 | WP_070071952 |
| *Acidimicrobium ferrooxidans* DSM 10331^T^ | Afer_0973 | ACU53912 |
| *Acidimicrobium ferrooxidans* DSM 10331^T^ | Afer_0974 | ACU53913 |
| *Acidithiobacillus caldus* SM-1 (CGMCC 1.7296) | Atc_2344 | AEK58992 |
| *Acidithiobacillus caldus* SM-1 (CGMCC 1.7296) | Atc_2346 | AEK58994 |
| *Acidithiobacillus ferrivoran*s SS3 (DSM 17398) | Acife_2468 | AEM48562 |
| *Acidithiobacillus ferrivoran*s SS3 (DSM 17398) | Acife_2470 | AEM48564 |
| *Acidithiobacillus ferrooxidans* ATCC 23270^T^ | AFE_2547 | ACK79688 |
| *Acidithiobacillus ferrooxidans* ATCC 23270^T^ | AFE_2549 | ACK78498 |
| *Acidithiobacillus ferrooxidans* ATCC 53993 | Lferr_2175 | ACH84379 |
| *Acidithiobacillus ferrooxidans* ATCC 53993 | Lferr_2177 | ACH84381 |
| *Acidithiobacillus thiooxidans* ATCC 19377^T^ | ATHIO_RS0101750 | WP_010637288 |
| *Acidithiobacillus thiooxidans* ATCC 19377^T^ | ATHIO_RS0101760 | WP_010637291 |
| *Atererythrobacter* *epoxidivorans* CGMCC 1.7731^T^ | AMC99_00773 | WP_061922963 |
| *Aquifex aeolicus* VF5 | aq_402 | O66720 |
| *Chloroherpeton thalassium* ATCC 35110^T^ | Ctha_0140 | ACF12611 |
| *Defluviimonas* *indica* DSM 24802^T^ | SAMN05444006_102168 | SDW24616 |
| *Defluviimonas* sp. 20V17 | U879_RS07770 | WP_035841171 |
| *Escherichia coli* K12 | SF31_19040 | AKD93032 |
| *Ectothiorhodospira haloalkaliphila* ATCC 51935^T^ | ECTHA_RS0104455 | WP_025282108 |
| *Ectothiorhodospira haloalkaliphila* ATCC 51935^T^ | ECTHA_RS0104445 | WP_025282106 |
| *Ectothiorhodosinus mongolicus* M9^T^ (DSM 15479^T^) | B0B04_RS03030 | [WP_076754807](https://www.ncbi.nlm.nih.gov/protein/1140905876) |
| *Ectothiorhodosinus mongolicus* M9^T^ (DSM 15479^T^) | B0B04_RS03040 | WP_076754809 |
| *Ectothiorhodospira marina* DSM 241^T^ | SAMN05444515_102239 | WP_090251068 |
| *Ectothiorhodospira marina* DSM 241^T^ | SAMN05444515_102241 | WP_090251072 |
| *Ectothiorhodospira mobilis* DSM 4180 | SAMN05421721_10350 | WP_090483756 |
| *Ectothiorhodospira mobilis* DSM 4180 | SAMN05421721_10352 | WP_090483758 |
| *Ectothiorhodospira* sp. PHS-1 | ECTPHS_RS10605 | WP_008932750 |
| *Ectothiorhodospira* sp. PHS-1 | ECTPHS_RS10615 | WP_008932752 |
| *Ferrithrix thermotolerans* DSM 19514^T^ | BUA24_RS08735 | WP_072791444 |
| *Ferrithrix thermotolerans* DSM 19514^T^ | BUA24_RS08740 | WP_072791447 |
| *Gordonia rhizosphera* NBRC 16068^T^ | GORHZ_RS13480 | NZ_BAHC01000000 |
| *Halorhodospira halochloris* str. A (DSM 1059^T^) | M911_11200 | AHK79630 |
| *Halorhodospira halochloris* str. A (DSM 1059^T^) | M911_11210 | AHK79632 |
| *Hoeflea* sp. BRH_c9 | VR78_06480 | KJS17053 |
| *Hydrogenivirga* sp. 128-5-R1-1 | HG1285_RS14255 | WP_008286388 |
| *Hydrogenobacter thermophilus* TK-6^T^ (DSM 6534^T^) | HTH_RS09400 | WP_012964498 |
| *Hydrogenobacter thermophilus* TK-6^T^ (DSM 6534^T^) | HTH_RS09415 | WP_012964501 |
| *Hydrogenobaculum* sp. HO | HydHO_1068 | AGG15383 |
| *Hydrogenobaculum* sp. HO | HydHO_1071 | AGG15386 |
| *Hydrogenobaculum* sp. Y04AAS1 | HY04AAS1_RS05425 | WP_012514115 |
| *Hydrogenobaculum* sp. Y04AAS1 | HY04AAS1_RS05440 | WP_012514118 |
| *Hyphomicrobium denitrificans* ATCC 51888^T^ | Hden_0696 | ADJ22516 |
| *Hyphomicrobium denitrificans* ATCC 51888^T^ | Hden_2793 | ADJ24589 |
| *Hyphomicrobium* sp. GJ21 | HYPGJ_RS10105 | WP_046847083 |
| *Kyrpidia tusciae* DSM 2912^T^ | Btus_2489 | ADG07149 |
| *Kyrpidia tusciae* DSM 2912^T^ | Btus_2490 | ADG07150 |
| *Metallosphaera cuprina* Ar-4^T^ (JCM 15769^T^) | Mcup_0662 | AEB94767 |
| *Metallosphaera sedula* DSM 5348^T^ | Msed_1570 | ABP95725 |
| *Rhodobacter aestuarii* JA296^T^ (JCM 144887^T^) | BW967_RS14430 | WP_076485799 |
| *Rhodobacter* sp. SW2 | RSW2DRAFT_RS16455 | WP_008033037 |
| *Saccharomonospora marina* XMU15 (DSM 45390^T^) | SACMADRAFT_RS09400 | WP_009153571 |
| *Saccharomonospora marina* XMU15 (DSM 45390^T^) | SACMADRAFT_RS09445 | WP_009153580 |
| *Saccharomonospora marina* XMU15 (DSM 45390^T^) | SACMADRAFT_RS09450 | WP_009153581 |
| *Sulfobacillus acidophilus* DSM 10332^T^ | Sulac_1389 | AEW04886 |
| *Sulfobacillus acidophilus* DSM 10332^T^ | Sulac_1390 | AEW04887 |
| *Sulfobacillus acidophilus* TPY | TPY_3525 | AEJ41677 |
| *Sulfobacillus acidophilus* TPY | TPY_3526 | AEJ41678 |
| *Sulfobacillus thermosulfidooxidans* DX | BFX05_RS00170 | WP_0760055821 |
| *Sulfobacillus thermosulfidooxidans* DX | BFX05_RS00175 | WP_020373190 |
| *Sulfolobus acidocaldarius* DSM 639^T^ | Saci_0349 | AAY79765 |
| *Sulfolobus acidocaldarius* DSM 639^T^ | Saci_0350 | AAY79766 |
| *Sulfolobus “islandicus”* M.14.25 | M1425_1122 | ACP37884 |
| *Sulfolobus solfataricus* P2 (DSM 1617) | SSO1105 | AAK41362 |
| *Sulfolobus tokodaii* str. 7 (DSM 16993^T^) | STK_18940 | BAB66988 |
| *Sulfolobus tokodaii* str. 7 (DSM 16993^T^) | STK_18950 | BAB66989 |
| *Sulfuricella denitrificans* skB26^T^ (DSM 22764^T^) | SCD_RS03980 | WP_021035773 |
| *Thermithiobacillus tepidarius* DSM 3134^T^ | G579_RS0105280 | WP_028989345 |
| *Thermithiobacillus tepidarius* DSM 3134^T^ | G579_RS0105270 | WP_038018313 |
| *Thermocrinis albus* DSM 14484^T^ | THAL_RS06810 | WP_012992376 |
| *Thermocrinis albus* DSM 14484^T^ | THAL_RS06825 | WP_012992379 |
| *Thermocrinis minervae* DSM 19557^T^ | SAMN05444391_1184 | SHK48708 |
| *Thermocrinis minervae* DSM 19557^T^ | SAMN05444391_1187 | SHK48788 |
| *Thermocrinis jamiesonii* GBS^T^ (DSM 27162^T^) | K217_RS0105850 | WP_029552192 |
| *Thermocrinis jamiesonii* GBS^T^ (DSM 27162^T^) | K217_RS0105865 | WP_029552195 |
| *Thermomicrobium roseum* DSM 5159^T^ | trd_0143 | ACM05628 |
| *Thermomicrobium roseum* DSM 5159^T^ | trd_0144 | ACM04497 |
| *Thermomicrobium roseum* DSM 5159^T^ | trd_0157 | ACM04821 |
| *Thioalkalivibrio nitratireducens* DSM 14787^T^ | TVNIR_3241 | AGA34878 |
| *Thioalkalivibrio nitratireducens* DSM 14787^T^ | TVNIR_3243 | AGA34880 |
| *Thioalkalivibrio* sp. AKL11 | D574_RS0111990 | WP_083916433 |
| *Thioalkalivibrio* sp. AKL11 | D574_RS0112000 | WP_018941302 |
| *Thioalkalivibrio* sp. ALJ3 | C935_RS0110705 | WP_081616601 |
| *Thioalkalivibrio* sp. ALJ3 | C935_RS0110715 | WP_018862495 |
| *Thioalkalivibrio* sp. ALMg13-2 | F618_RS0106170 | WP_077276997 |
| *Thioalkalivibrio* sp. ALMg13-2 | F618_RS0106180 | WP_018168416 |
| *Thioalkalivibrio* sp. K90mix | TK90_0638 | ADC71153 |
| *Thioalkalivibrio* sp. K90mix | TK90_0640 | ADC71155 |
| *Thioalkalivibrio sulfidiphilus* HL-EbGr7 | Tgr7_2208 | ACL73288 |
| *Thioalkalivibrio sulfidiphilus* HL-EbGr7 | Tgr7_2210 | ACL73290 |
| *Thioalkalivibrio versutus* | TVD_10610 | AKJ95782 |
| *Thioalkalivibrio versutus* | TVD_10620 | AKJ95783 |
| *Thiohalorhabdus denitrificans* HL 19 (DSM 15699^T^) | BLP36_RS08615 | WP_054965387 |
| *Thiohalorhabdus denitrificans* HL 19 (DSM 15699^T^) | BLP36_RS08625 | WP_054965385 |
| *Thiohalospira halophila* DSM 15071^T^ | SAMN05660831_02558 | SFD89889 |
| *Thiohalospira halophila* DSM 15071^T^ | SAMN05660831_02560 | SFD89950 |
| *Thiorhodospira sibirica* ATCC 700588^T^ | ThisiDRAFT_2440 | EGZ43482 |
| *Thiorhodospira sibirica* ATCC 700588^T^ | ThisiDRAFT_1533 | EGZ46119 |
