# Supplementary material for: Lipoate-binding proteins and specific lipoate-protein ligases in microbial sulfur oxidation reveal an atpyical role for an old cofactor
Source: eLife. 2018 Jul 13;7:e37439. doi: 10.7554/eLife.37439 (PMC6067878; doi:10.7554/eLife.37439)
Supplement: Supplementary file 1. — Table listing the strains and plasmids used in this study. [file elife-37439-supp1.docx]

**Table S1. Bacterial strains**

| **Strains** | **Relevant genotype** | **Reference or source** |
| --- | --- | --- |
| *Thiorhodospira sibirica* ATCC 700588^T^ | Wild type | (*Bryantseva et al., 1999*) |
| *Thioalkalivibrio* sp. K90 mix | Wild type | (*Muyzer et al., 2011*) |
| *Hyphomicrobium denitrificans* X^T^  (ATCC 51888) | Wild type | (*Urakami et al., 1995*) |
| *H. denitrificans* Sm200 | Sm^r^, spontaneous streptomycin-resistant mutant of *H. denitrificans* ATCC 51888^T^ | (*Koch and Dahl, 2018*) |
| *H. denitrificans* ∆*lbpA* | Sm^r^, *in-frame* deletion of *lbpA2* (Hden_0696) in *H. denitrificans* Sm200 | This work |
| *H. denitrificans* ∆*hdr* | Sm^r^, Tc^r^, Hden_0691-Hden_0692::ΩTet (in Sm200) | (*Koch and Dahl, 2018*) |
| *H. denitrificans hdr*^+^ | Tc^r^, Kmr^r^ *H. denitrificans* wt with plasmid pBBR1p264HdenHdrTet | (*Koch and Dahl, 2018*) |
| *Escherichia coli* DH5α | *fhuA2 Δ(argF-lacZ)U169 phoA glnV44 Φ80 Δ(lacZ)M15 gyrA96 recA1 relA1 endA1 thi-1 hsdR17* | (*Hanahan, 1983*) |
| *E. coli* NEB 10β | Δ(*ara-leu) 7697 araD139 fhuA* Δ*lacX74 galK16 galE15 e14-* ϕ*80*d*lacZ*Δ*M15 recA1 relA1 endA1 nupG rpsL* (Str^r^) *rph spoT1* Δ*(mrr-hsdRMS-mcrBC)* | New England Biolabs |
| *E. coli* BL21 DE3 | *B dcm ompT hsdS(r_B_^-^m_B_^-^) gal* | Novagen |
| *E. coli* MG1655 | *rph-1* | CGSC^a^ |
| *E. coli* QC146 | Δ*lplA*::*FRT* Δ*lipB*::*FRT* | (*Christensen and Cronan, 2009*) |
| *E. coli* DK574 | *metB1 relA1 spoT1* λ^-^ λ^r^ *gyrA216 panD2 zad-220*::Tn*10 F^-^* with pMS421 and pMR19 | (*Keating et al., 1995*) |
| *E. coli* BL21 (DE3) Δ*iscR* | F^-^ *ompT* *hsdS*_B_(r_B_^-^m_B_^-^) *gal dcm* Δ*iscR*::Kan (DE3) | (*Akhtar and Jones, 2008*) |
| *Bacillus subtilis* JH642 | *trpC2 pheA1* | Lab stock |
| *B. subtilis* NM20 | JH642 *gcvH*::Km | (*Christensen et al., 2011*) |

^a^CGSG denotes the *E. coli* Genetic Stock Center

**References**

Akhtar MK, Jones PR. 2008. Deletion of *iscR* stimulates recombinant clostridial Fe-Fe hydrogenase activity and H_2_-accumulation in *Escherichia coli* BL21(DE3). *Applied Microbiology and Biotechnology* **78**:853-862. doi: 10.1007/s00253-008-1377-6, PMID: 18320190

Bryantseva IA, Gorlenko VM, Kompantseva EI, Imhoff JF, Sling J, Mityushina L. 1999. *Thiorhodospira sibirica* gen. nov., sp. nov., a new alkaliphilic purple sulfur bacterium from a Siberian soda lake. *International Journal of Systematic Bacteriology* **49**:697-703. doi: 10.1099/00207713-49-2-697, PMID: 10319493

Christensen QH, Cronan JE. 2009. The *Thermoplasma acidophilum* LplA-LplB complex defines a new class of bipartite lipoate-protein ligases. *Journal of Biological Chemistry* **284**:21317-21326. doi: 10.1074/jbc.M109.015016, PMCID: PMC2755856

Christensen QH, Martin N, Mansilla MC, de Mendoza D, Cronan JE. 2011. A novel amidotransferase required for lipoic acid cofactor assembly in *Bacillus subtilis*. *Molecular Microbiology* **80**:350-363. doi: 10.1111/j.1365-2958.2011.07598.x, PMCID: PMC3088481

Hanahan D. 1983. Studies on transformation of *Escherichia coli* with plasmids. *Journal of Molecular Biology* **166**:557-580. doi: PMID: 6345791

Keating DH, Carey MR, Cronan JE, Jr. 1995. The unmodified (apo) form of *Escherichia coli* acyl carrier protein is a potent inhibitor of cell growth. *Journal of Biological Chemistry* **270**:22229-35. doi: PMID: 7673201

Koch T, Dahl C. 2018. A novel bacterial sulfur oxidation pathway provides a new link between the cycles of organic and inorganic sulfur compounds. *ISME Journal* **submitted**doi:

Muyzer G, Sorokin DY, Mavromatis K, Lapidus A, Foster B, Sun H, Ivanova N, Pati A, D'Haeseleer P, Woyke T, Kyrpides NC. 2011. Complete genome sequence of *Thioalkalivibrio* sp. K90mix. *Standards in Genomic Sciences* **5**:doi:10.4056/sigs.2315092. doi: 10.4056/sigs.2315092, PMCID: PMC3368412

Urakami T, Sasaki J, Suzuki KI, Komagata K. 1995. Characterization and description of *Hyphomicrobium denitrificans* sp. nov. *International Journal of Systematic Bacteriology* **45**:528-532. doi: Doi 10.1099/00207713-45-3-528, PMCID: PMC183213
