# Supplementary material for: Lipoate-binding proteins and specific lipoate-protein ligases in microbial sulfur oxidation reveal an atpyical role for an old cofactor
Source: eLife. 2018 Jul 13;7:e37439. doi: 10.7554/eLife.37439 (PMC6067878; doi:10.7554/eLife.37439)
Supplement: Supplementary file 2. — Table listing the primer and plasmid names and properties used in this study. [file elife-37439-supp2.docx]

**Table S2. Primers and plasmids**

| **Primers or plasmids** | **Relevant genotype, description or sequence** | **Reference or source** |
| --- | --- | --- |
| **Primers** |  |  |
| ThisiDRAFT_1533 (*lbpA1*) Strep fw | ACAAC**CATATG**AGCGCTTGGAGCCACCCGCAGTTCGAAAAAGATGACTGTAAC (NdeI) | This study |
| ThisiDRAFT_1533 (*lbpA1*) rev | CGAGC**GAATTC**TTATGCCGTGC | This study |
| Hden_0696 *(lbpA2*) Strep fw | GAGTGA**CATATG**AGCGCTTGGAGCCACCCGCAGTTCGAAAAACCAGTCGTGAAGGGATGCAATCTA (NdeI) | This study |
| Hden_0696 *(lbpA2*) rev | GCCGT**GAATTC**TCAGCCGCAACCTGCGAAA (EcoRI) | This study |
| TK90_0642 (*lplA1,* single domain) fw | CTGCC**GAATTC**ATGTCTGAACGCGATCA (EcoRI) | This study |
| TK90_0642 (*lplA1,* single domain) rev | GCGCAA**TCTAGA**TTCATCCCGCTGATTCCT (XbaI) | This study |
| Hden_0686 (*lplA,* single domain) fw | TCGAGACGGGTG**CCATGG**CATGAGTATAG (NcoI) | This study |
| Hden_0686 (*lplA,* single domain) rev BAD | CCGAGA**TCTAGA**TCATGGCCTCAGCGTC (XbaI) | This study |
| Hden_0686 (*lplA,* single domain) Strep fw | TGATCT**CATATG**AGCGCTTGGAGCCACCCGCAGTTCGAAAAAAGTATAGGGCGGCGCGCATT (NdeI) | This study |
| Hden_0686 (*lplA,* single domain) rev pET | TGACCGA**GAATTC**CTTCATGGCCTCAG (EcoRI) | This study |
| TK90_0638 (*lbpA1*) fw | TTTTTT**CATATG**GACTGCAACGGTTGC (NdeI) | This study |
| TK90_0638 (*lbpA1*) rev | TTTTTT**CTCGAG**CTATCGCAGCTTGCGCTG (XhoI) | This study |
| TK90_0640 (*lbpA2*) fw | TTTTTT**CATATG**GGCGCAGTACGGGGT(NdeI) | This study |
| TK90_0640 (*lbpA2*) rev | TTTTTT**GGATCC**TTAGCAGCCGCCGAAAC (BamHI) | This study |
| XC257/ XC259 forward (with RBS, *HindIII*) for TsLbpA1 and HdLbpA2 | TCTAT**AAGCTT**AAAACCATTATATTAGGAGGAAATAACATGAGCGCTTGGAGCCACCC (HindIII) | This study |
| XC258 reverse (*SphI*) for TsLbpA1 | TCTAT**GCATGC**TTATGCCGTGCGGGTGCAGTG (SphI) | This study |
| XC260 reverse (*SphI*) for HdLbpA2 | TCTAT**GCATGC**TCAGCCGCAACCTGCGAAACCTTC (SphI) | This study |
| Fwd5'_∆lbpA | AAAA**GCATGC**CACCAAGGGACGGCTCGCT (SphI) | This study |
| Rev5'_∆lbpA | TAAGAGCGGCCGTAGAAGCTCA**CCCGGG**CATAGTTCACTCCGATATTG (SmaI) | This study |
| Fwd3'_∆lbpA | AAAA**TCTAGA**GCGATCGCTCGATGGAAAA (XbaI) | This study |
| Rev3'_∆lbpA | CAATATCGGAGTGAACTATG**CCCGGG**TGAGCTTCTACGGCCGCTCTTA (SmaI) | This study |
| **Plasmids** |  |  |
| pET22b | Ap^r^ | Novagen |
| pHP45Ω-Tc | Ap^r^, Tc^r^ | (*Fellay et al., 1987*) |
| pk18*mobsacB* | Km^r^, Mob^+^, *sacB*, *oriV*, *oriT*, *lacZ*α | (*Schäfer et al., 1994*) |
| pk18*mobsacB*∆lbpA2 | Km^r^, 2.02 kB SOE PCR fragment cloned into pk18*mobsac*B using SphI and XbaI restriction sites | This study |
| pk18*mobsacB*∆lbpA2Tc | Tc^r^, pHP45Ω-Tc tetracycline cassette excised with SmaI and inserted into pk18*mobsacB*∆lbpA2 using BglII and Klenow fill in | This study |
| pBBR1p264HdenHdrTet | Km^r^, Tc^r^, genes Hden_0688 to Hden_0696 and pHP45Ω-Tc tetracycline cassette in pBBR1p264 | (*Koch and Dahl, 2018*) |
| pBAD22A | Ap^r^ | (*Guzman et al., 1995*) |
| pdr-111-amyE-hyper-SPANK | Ap^r^, Spec^r^, IPTG-inducible shuttle vector, *5-amyE*, *3-amyE*, P_hyper spank_ | Gift by David Rudner |
| pdr-111-amyE-hyper-SPANK-*TsLbpA1* | Ap^r^, Spec^r^, SphI-HindIIIfragment of PCR amplified ThisiDRAFT_1533 (*lbpA1*) with ribosome binding site added in pdr-111-amyE-hyper-SPANK | This study |
| pdr-111-amyE-hyper-SPANK-HdLbpA2 | Ap^r^, Spec^r^, SphI-HindIII fragment of PCR amplified Hden_0696 (*lbpA2*) with ribosome binding site added in pdr-111-amyE-hyper-SPANK | This study |
| pET-*Ts*LbpA1-Strep (ThisiDRAFT_1533) | Ap^r^, N-terminal Strep-tag, NdeI-EcoRI fragment of PCR amplified ThisiDRAFT_1533 (*lbpA1*) in pET22b | This study |
| pET-*Hd*LbpA2-Strep (Hden_0696) | Ap^r^, N-terminal Strep-tag, NdeI-EcoRI fragment of PCR amplified Hden_0696 (*lbpA2*) in pET22b | This study |
| pBAD-*Tk90*LplA (TK90_0642) | Ap^r^, EcoRI-XbaI fragment of PCR amplified TK90_0642 (*lplA*) in pBAD | This study |
| pBAD-*Hd*LplA (Hden_0686) | Ap^r^ NcoI-XbaI fragment of PCR amplified Hden_0686 (*lplA*) in pBAD | This study |
| pET—*Hd*LplA-Strep (Hden_0686) | Ap^r^, NdeI-EcoRI fragment of PCR amplified Hden_0686 (*lplA*) in pET22b | This study |
| pET-*TK90*LbpA1 (TK90_0638) | Ap^r^, NdeI-XhoI fragment of PCR amplified TK90_0638 (*lbpA1*) in pET22b | This study |
| pET-*TK90*LbpA2 (TK90_0640) | Ap^r^, NdeI-BamHI fragment of PCR amplified TK90_0640 (*lbpA2*) in pET15b | This study |
| pET-*Tk90*LplA (TK90_0642) | Km^r^, NdeI-BamHI fragment of PCR amplified TK90_0642 (*lplA*) in pET28b | This study |

**References**

Fellay R, Frey J, Krisch HM. 1987. Interposon mutagenesis of soil and water bacteria: a family of DNA fragments designed for in vivo insertional mutagenesis of Gram-negative bacteria. *Gene* **52**:147-154. doi: 10.1016/0378-1119(87)90041-2, PMID: 3038679

Guzman L, Belin D, Carson MJ, Beckwith J. 1995. Tight regulation, modulation, and high-level expression by vectors containing the arabinose P_BAD_ promotor. *Journal of Bacteriology* **177**:4121-4130. doi: PMID: 7608087

Koch T, Dahl C. 2018. A novel bacterial sulfur oxidation pathway provides a new link between the cycles of organic and inorganic sulfur compounds. *ISME Journal* **submitted**

Schäfer A, Tauch A, Jäger W, Kalinowski J, Thierbach G, Pühler A. 1994. Small mobilizable multi-purpose cloning vectors derived from the *Escherichia coli* plasmids pK18 and pK19: selection of defined deletions in the chromosome of *Corynebacterium glutamicum*. *Gene* **145**:69-73. doi: PMID: 8045426
